# Risk of death during acute infection is accelerating across diverse host-pathogen systems and consistent with multiple models of host-pathogen interaction

**DOI:** 10.1128/msphere.00953-24

**Published:** 2025-04-28

**Authors:** Tim O'Sullivan, Canan Karakoç, Kristofer Wollein Waldetoft, Sam P. Brown

**Affiliations:** 1 School of Biological Sciences, Georgia Institute of Technology 123387 , Atlanta, Georgia, USA; 2 Center for Microbial Dynamics and Infection, Georgia Institute of Technology 1372, Atlanta, Georgia, USA; 3 Torsby Hospital 174456, Torsby, Varmland County, Sweden; NC State University, Raleigh, North Carolina, USA

**Keywords:** mortality, death, age of infection, meta-analysis, virulence, within-host dynamics

## Abstract

Infectious diseases remain a major cause of global mortality, yet basic questions concerning the relationship between within-host processes governing pathogen burden (pathogen replication, immune responses) and population-scale (epidemiological) patterns of mortality remain obscure. We use a structured literature review to leverage the extensive biomedical data generated by controlled host infections to address the epidemiological question of whether infection-induced mortality is constant, accelerating, or follows some other pattern of change and to infer the within-host mechanistic basis of this pattern. We show that across diverse lethal infection models, the risk of death increases approximately exponentially in time since infection, in a manner phenomenologically similar to the dynamics of all-cause death. We further show that this pattern of accelerating risk is consistent with multiple alternate mechanisms of pathogen growth and host-pathogen interaction, underlining the limitations of current experimental approaches to connect within-host processes to epidemiological patterns. We review critical experimental questions that our work highlights, requiring additional non-invasive data on pathogen burden throughout the course of infection.

**IMPORTANCE:**

Here, we ask a simple question: what are the dynamics of pathogen-induced death? Death is a central phenotype in both biomedical and epidemiological infectious disease biology, yet very little work has attempted to link the biomedical focus on pathogen dynamics within a host and the epidemiological focus on populations of infected hosts. To systematically characterize the dynamics of death in controlled animal infections, we analyzed 209 data sets spanning diverse lethal animal infection models. Across experimental models, we find robust support for an accelerating risk of death since the time of infection, contrasting with conventional epidemiological models that assume a constant elevated risk of death. Using math models, we show that multiple processes of growth and virulence are consistent with accelerating risk of death, and we end with a discussion of critical experiments to resolve how within-host biomedical processes map onto epidemiological patterns of disease.

## INTRODUCTION

Infectious disease research operates across multiple scales, from cell biologists studying the molecular interactions between pathogen and host cells to epidemiologists studying the trajectories of infection case counts through populations of hosts. The breadth of infection research is a testament to the enduring importance of infections to humanity and provides an incredible resource to infection researchers working across these scales. Yet the specialization of research has led to a relative disconnect between the within-host focus of biomedical research and the among-host focus of epidemiological research. In particular, we focus on a disconnect between analyzing a key infection outcome—life or death.

In the biomedical tradition, the study of mortality focuses on establishing pathogen causal mechanisms of host damage and methods to control infection. A primary strategy to identify mechanisms of host damage [virulence factors (1)] or therapeutics is to conduct infection screens using well-controlled experimental infections of animal models. These screens are typically conducted across genetic knockout and chemical libraries to identify genes (virulence factors) or compounds (antimicrobials) of interest. Thanks to this thriving tradition, we now have extensive databases on pathogen virulence factors (2–4) and chemical interactions, accompanied in many cases by detailed molecular mechanisms underpinning their mode of action on host cells or tissues, their regulatory control, delivery, host immune responses, etc.

In contrast, the epidemiological approach focuses on describing, interpreting, and predicting the trajectory of infections on the host population scale. This work’s critical focus is how pathogen transmission, host recovery, and infection-induced mortality combine to govern whether an infection expands or contracts on a population scale. Infectious disease epidemiology is characterized by a solid mathematical modeling tradition applied to clinical case-report data on human infections (5). The dominant modeling traditions treat pathogen-induced mortality as a constant (elevated above background mortality) throughout the duration of infection. Undoubtedly, this assumption simplifies the analysis and allows for a focus on the transmission dynamics of infection and recovery (5–9). Yet, in the few contexts where high-resolution temporal data on infection-induced human mortality is available on a population scale (notably for sepsis), there is evidence that the risk of death is increasing with time (10, 11).

In this paper, we seek to leverage the extensive published biomedical data generated by controlled host infections to address the epidemiological question of whether infection-induced mortality is constant, accelerating, or follows some other pattern of change. We further explore how a diverse set of simple phenomenological models of within-host pathogen growth can give rise to an accelerating risk of death. We find that across diverse infection models, the risk of death generally accelerates exponentially in time since infection. We further show that this pattern of accelerating risk is consistent with multiple alternate mechanisms of pathogen growth and host interaction, underlining the limitations of current experimental approaches to connect within-host processes to epidemiological patterns.

## RESULTS

To assess the dynamics of infection-induced mortality across experimental host-pathogen systems, we performed a structured literature search of the Web of Science and EBSCO Medline databases. Applying the following search term (experiment* OR animal model) AND (infectio* OR viru* OR pathogen OR parasite) AND ("survival analysis" OR "survival assay" OR "survival rate" OR "mortality rate" OR "death rate" OR "time to death") resulted in over 6,000 papers between 1991 and 2024. We added 11 relevant papers that did not appear in this search (indicated in Table S2). An initial manual review of the title and the abstract resulted in 252 papers that are not evidently off-topic. These 252 papers were then screened in detail, with individual experiments within each paper being rejected if any of the following conditions held: (i) less than a minimum of five observations in time, (ii) less than a minimum of five hosts per treatment, (iii) immune-modifying treatments, (iv) non-wildtype pathogen (e.g., virulence factor knockout strain), (v) infection treatment (e.g., antibiotics), (vi) no defined pathogen infection dose or exposure, and (viii) less than 80% of the host died after infection. This process resulted in survival data for 209 experimental data sets from 82 papers (Table S2). We separately recorded all individual qualifying experiments for each qualifying paper, extracting data from tables or graphs (using WebPlotDigitizer [12]) when the raw data were unavailable. Of these 82 papers, 39 reported raw data with or without replicates (replicates averaged prior to analysis). The remaining 43 papers reported survival probability estimated with the Kaplan-Meier method (13). Reporting survival probability is common, especially in clinical studies with mammals, when the subjects die or are removed from the experiments. Therefore, we also considered these probability data while reporting the difference in methodology (see Materials and Methods).

**Fig 1:**
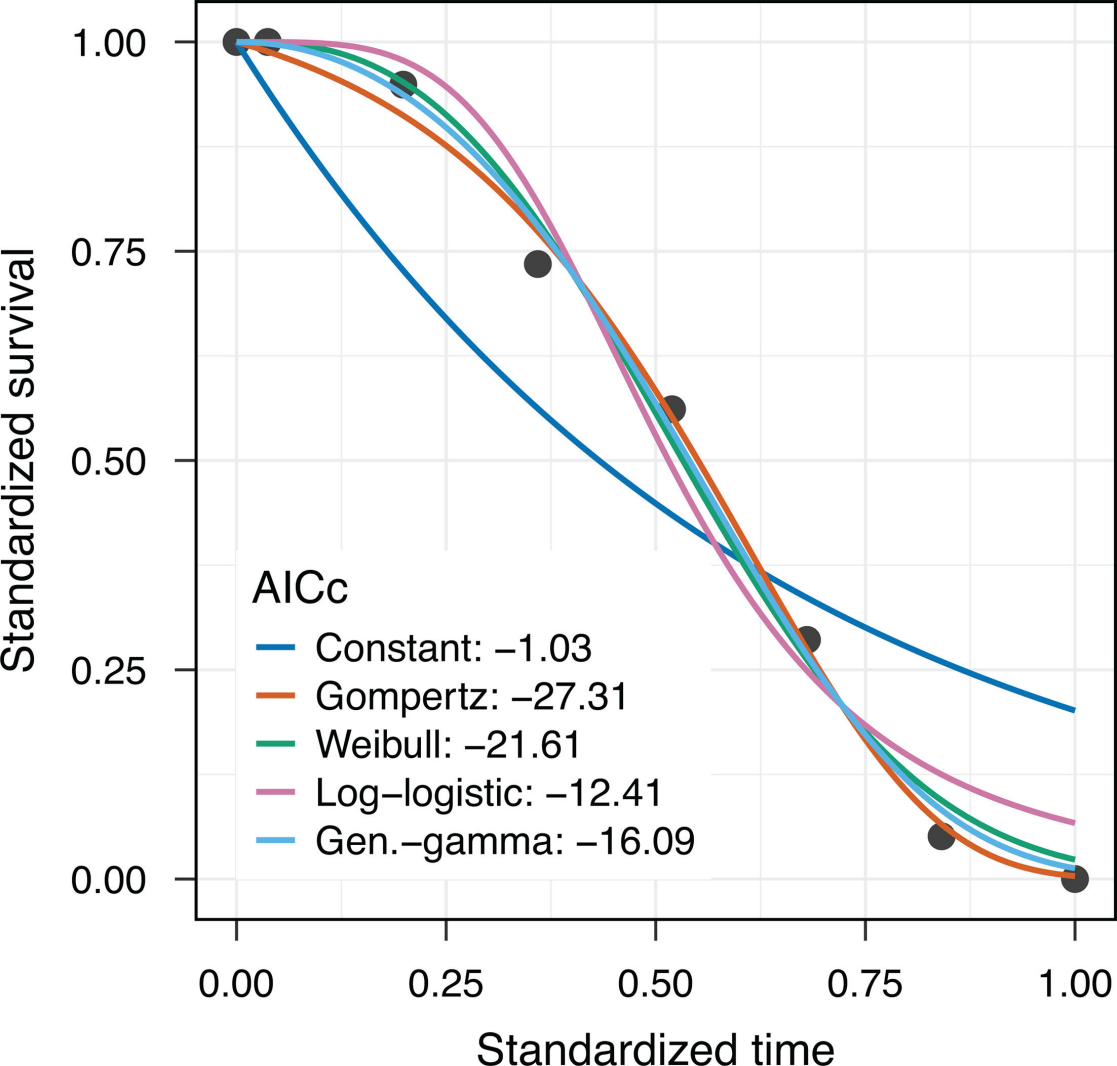
Survival data for the zebrafish*-Pseudomonas aeruginosa* model system indicate the failure of a constant mortality assumption. Black circles represent experimental data on the proportional survival of zebrafish (host *N* = 18, across three replicated experiments) infected with 1 × 10^8^ cfu/mL *Pseudomonas aeruginosa* using immersion challenge method (i.e., bathing the fish in a pathogen suspension) (14). Survivorship was tracked over 96 hours (here and in other analyses rescaled to a unit interval). The blue line represents a constant mortality assumption (an exponential model fit). The green line represents an exponentially increasing mortality assumption (Gompertz model fit). The other lines represent alternate models of non-constant mortality (generalized gamma, log-logistic, and Weibull model fits). Model comparisons are made with the corrected Akaike Information Criterion (AICc) assessing predictive accuracy, with lower AICc scores indicating a more parsimonious model fit (15, 16). All model fits were made using the Levenberg-Marquardt non-linear least squares algorithm, implemented in R (17, 18).

Our structured literature review returned a phylogenetically diverse range of hosts and pathogens. The experimental hosts spanned mice, fruit flies (*Drosophila* sp.), other insects (grasshoppers, honeybees, mosquitoes, bat flies, *Triatoma*), nematodes (*Caenorhabditis elegans*), and other invertebrates (starfish, shrimps, water fleas), vertebrates (zebrafish larvae, fish, mice, guinea pigs, chickens, primates), and seedlings. Our pathogens spanned DNA and RNA viruses, gram-negative and gram-positive bacteria, protozoan parasites, and fungi. Collectively, these infection models represent many different mechanisms of host immunity and pathogen virulence. Prior to surveying all of the data together, we begin with an illustrative example to orient our subsequent analyses (Fig. 1), featuring the proportion of surviving zebrafish after experimental infection with the bacterial pathogen *Pseudomonas aeruginosa* (data from reference 14).

To assess whether we see an accelerating risk of death over time since infection, we first fit a constant mortality model to capture the null hypothesis of constant risk [instantaneous mortality rate at a time *t* , *m*(*t*) = λ; Fig. 1 blue line]. On even a cursory examination, the survivorship data systematically deviate from the constant mortality fit, with higher survivorship early in the infection and lower survivorship late in the infection. In other words, the data indicate support for an increasing risk of death as the infection proceeds.

### Pathogen-induced death as a process of accelerated aging

This pattern of increasing instantaneous mortality or “hazard” through time is familiar from the aging literature. In a human context, the instantaneous risk of death rises approximately exponentially across the lifespan, doubling roughly every 7 years (19). In controlled animal aging experiments, the same statistical patterns are observed, with the absolute rates varying with the animal (20–23), their genetics (24), and the environment (25, 26). Working from this connection, we next decided to test one of the simplest and common models from the aging literature, the Gompertz (27, 28). Briefly, the two-parameter Gompertz mortality function defines the instantaneous rate of mortality $m\left(t\right)$ as an exponentially increasing function of t: ${m\left(t\right)=a*e}^{b*t}$. When b=0, the Gompertz recovers a constant mortality process at the rate a. Increasing *b* leads to an increasing acceleration of instantaneous mortality with time. Finally, for each data set, we also assessed other common models from the aging literature that can capture a variety of non-constant mortality trajectories (Weibull [29], log-logistic [30], and gamma distribution [31] summarized in Table 1).

**TABLE 1:** Instantaneous mortality functions [*m*(*t*)] used in this study and corresponding survival function [*S*(*t*)]

Mortality model	Mortality function [*m*(*t*)]	Survival function [*S*(*t*)]
Constant	m(t)=λ	$S\left(t\right)=e^{-\lambda t}$
Gompertz	$m\left(t\right)={ae}^{bt}$	${S\left(t\right)=e^{-}}^{\frac{a}{b}\left(e^{bt}-1\right)}$
Weibull	$m\left(t\right)=\frac{k}{\lambda }{\left(\frac{t}{\lambda }\right)}^{k-1}$	$S\left(t\right)=e^{-(\frac{t}{\lambda k})}$
Log-logistic	$m\left(t\right)=\frac{\beta }{\alpha }.\frac{{\left(\frac{t}{\alpha }\right)}^{\beta -1}}{{1+\left(\frac{t}{\alpha }\right)}^{\beta }}$	$S\left(t\right)=\frac{1}{1+{\left(\frac{t}{\alpha }\right)}^{\beta }}$
Generalized gamma distribution	$m\left(t\right)=-\frac{d}{dt}ln(S\left(t\right))$	$S\left(t\right)=\left({\left(-\frac{t}{\beta }\right)}^{\gamma }{\left(1+(-1){\left(\frac{t}{\beta }\right)}^{\gamma }\right)}^{\frac{1}{\alpha -1}}\right)$

To fit each distribution in Table 1 to our experimental data sets, we used the Levenberg-Marquardt nonlinear least squares method, implemented in R (21) (see Materials and Methods for details). Applying these more complex models to our initial exploratory data set (Fig. 1), we see improvements in fit, but at the expense of an additional one or two parameters. We used an information criterion approach to assess model fit while accounting for the number of parameters (15). Specifically, we use the Corrected Akaike Information Criterion (AICc) as it is more robust than AIC when applied to smaller data sets (32). Lower values of AICc translate to greater support for a specific model, revealing the Gompertz model to be the best-supported model for the specific example data set in Fig. 1.

**Fig 2:**
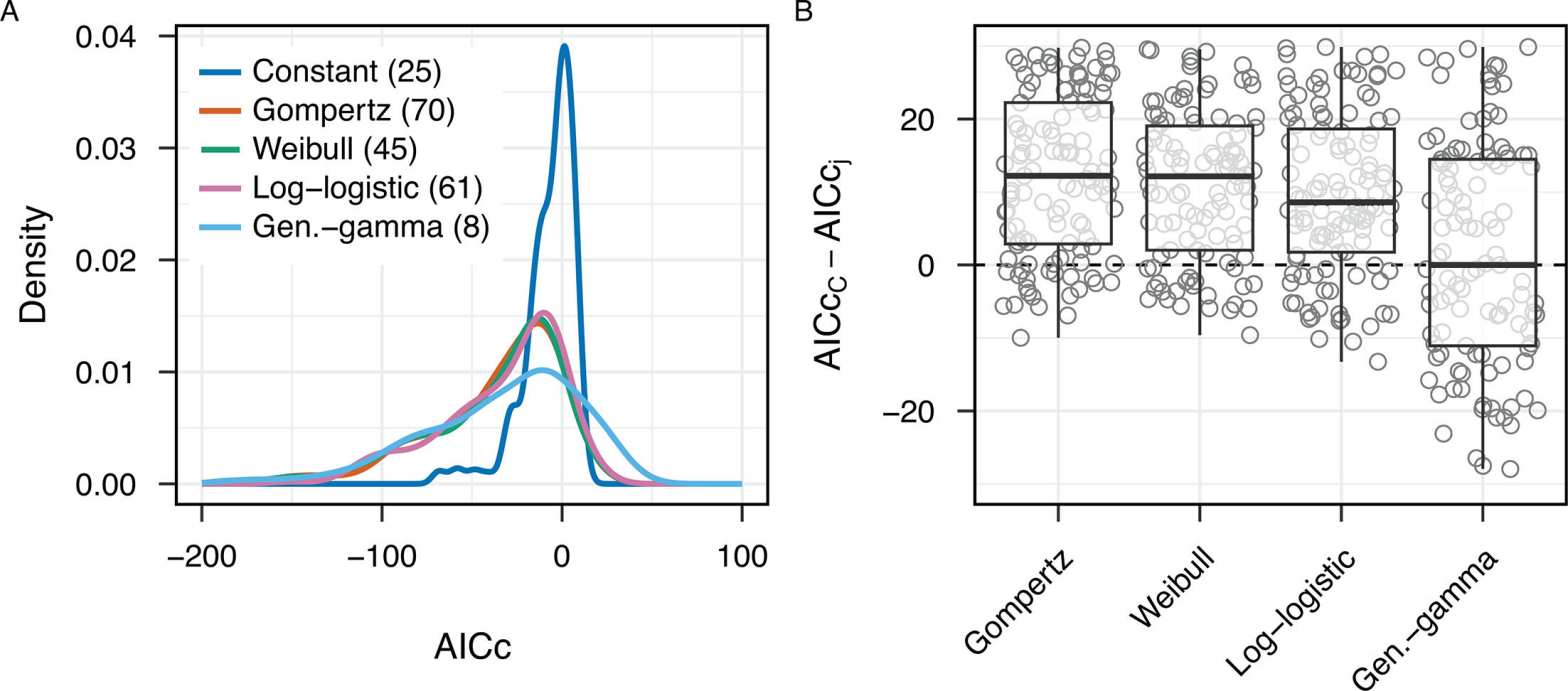
Survival data for 209 host-pathogen experimental models indicates the general failure of a constant mortality assumption. (**A**) Distributions of AICc scores across 209 studies for each mortality model (lower scores indicate more support). Note that positive values of AICc can arise when the complexity term (2*k*) and sample size correction [2*k*(*k* + 1)]/(*n − k* − 1) are greater in magnitude than the log likelihood term {AICc = 2*k* – 2 log(L) + [2*k*(*k* + 1)]/(*n−k−*1)}. While the absolute values of AICc are not meaningful, the relative differences between models guide interpretation. The bracketed inset numbers provide additional information on the number of cases where the specified model is the best-supported model (i.e., it has the lowest AICc score compared to all other models for a given data set). (**B**) Comparison of constant mortality model C with alternate models *j*: ∆_*Cj*_ = AICc*_C_* – AICc*_j_*. Positive ∆*_Cj_* values indicate the superiority of alternate models *j*. AICc data are truncated between AICc 30 and −30.

Using an information criterion approach to separate the exponential and Gompertz models for the data in Fig. 1 is an overkill, given the striking difference in model fit. Yet, it provides a currency to further interrogate comparative model performance across multiple data sets in our structured literature review (Table S2) and across multiple models of non-constant mortality (Table 1). Figure S1 illustrates survival data along with alternate model fits for each of the 209 experimental infection data sets from our structured literature review. Parameters and AICc of each model fit are provided in Table S3. In Fig. 2A, we summarize these analyses by plotting the distribution of AICc values across all data sets for each model in turn. From these distributions, we can see that, on average, the constant mortality model is less well supported (produces higher AICc scores) than the other models across the same ensemble of data sets. To support this claim, we built a linear mixed-effects model (with data set as random effect) which revealed a significant difference between constant and accelerating models on AICc (*χ*² = 277.22, *P* < 0.001), indicating that AICc performance varies across models. Bootstrap resampling (*R* = 1,000) confirmed the significance of this effect, with bias-corrected 95% confidence intervals for fixed effects consistently excluding zero.

To make specific model comparisons within each individual experimental data set, we assess differences in model performance for models *i* and *j* in each study as follows: ∆*_ij_* = AICc*_i_* – AICc*_j_*, where a positive ∆*_ij_* indicates greater support for model *j*, compared to model *i*. The magnitude of ∆*_ij_* provides information on the relative support for one model over another, given the data. Under an AIC approximation, we can use standard calculations for relative model likelihoods (16): ∆*_ij_* = 6 implies a 20-fold improvement for model *j* compared to *i*; ∆*_ij_* = 10 implies a 150-fold improvement, and ∆*_ij_* = 20 *a* >20,000-fold improvement. In Fig. 2B, we compare the constant mortality model C across all 209 experiments against each alternate non-constant model *j*, plotting ∆*_Cj_* = AICc*_C_* – AICc*_j_* for each alternate model, illustrating that the constant mortality model is generally inferior to the Gompertz and other accelerating models, while accelerating models have generally similar performance. This conclusion is further supported by additional comparisons of AICc across all models (Fig. S4) and an accompanying statistical analysis table (Table S1).

To assess the importance of mortality acceleration [$\frac{dm\left(t\right)}{d\left(t\right)}>0$], we make use of established mathematical thresholds defining positive and accelerating mortality for two of our defined functions. Specifically, for the Gompertz distribution, accelerating mortality is defined by b>0, and decelerating mortality by b<0 (b=0 implies constant mortality) (33, 34). For the Weibull function, k>1 defines accelerating mortality, while k<1 defines decelerating mortality (k=1 implies constant mortality) (35, 36). Our results show that for 98% of the Gompertz model fits (204 out of 209 data sets), the parameterization supported accelerating mortality (b>0). Strikingly, running the parallel “acceleration test” using the Weibull model resulted in an exact 100% match in the classification of accelerating data sets (the same 204 out of 209 studies were defined by k>1).

Taken together, our model comparisons illustrate the inferiority of the constant mortality model and the general equivalence of the Gompertz model to other non-constant mortality models. In light of this equivalence of a simple exponential mortality model with more complex models (Table 1 and Fig. 2B), we focus in the remainder of this paper on the Gompertz model as a simple phenomenological descriptor of mortality due to acute infection.

Our analysis, summarized in Fig. 2, illustrates the general failure of the constant mortality model and provides support for a simple model of exponentially increasing mortality. Yet we can also see substantial variation across individual experiments in the extent to which the Gompertz model is superior to the constant mortality model (∆_C*G*_ = AICc*_C_* – AICc*_G_*, first data column, Fig. 2B). Figure 2B illustrates that for some data sets, the Gompertz model G is substantially better than the constant mortality model C (∆*_CG_* > 20), whereas in other experiments, the difference is negligible (∆*_CG_* ~ 0).

**Fig 3:**
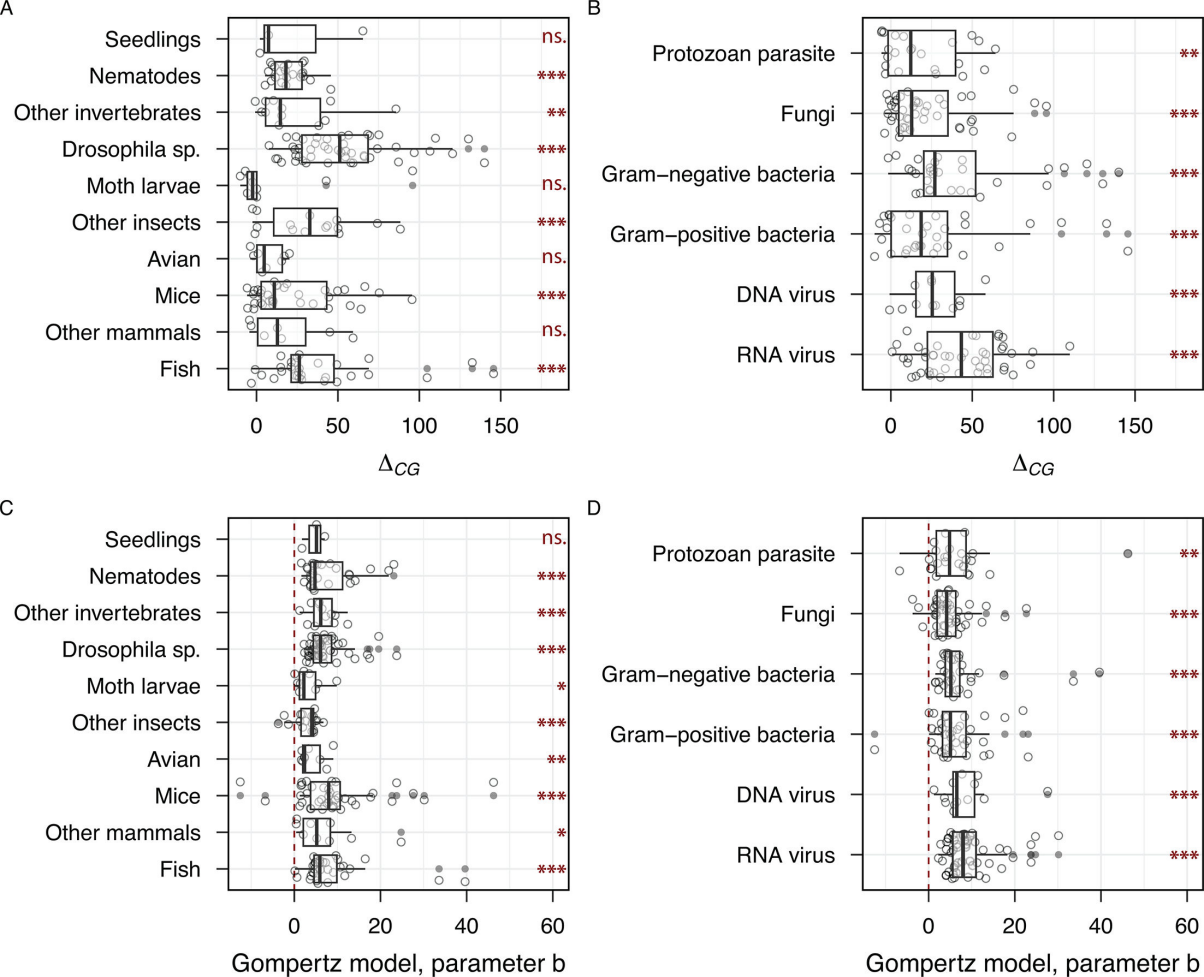
The accelerating risk model is supported across a broad range of host and pathogen taxa. (**A, B**) AICc differences (Δ*_CG_*), showing the relative support for the Gompertz model compared to the constant mortality model across (**A**) host and (**B**) pathogen taxa. (**C, D**) Estimates of the Gompertz model parameter *b* for each data set grouped by (**C**) host and (**D**) pathogen taxa. Positive values of *b* indicate accelerating mortality. Statistical significance of whether Δ*_CG_* and *b* significantly differ from zero was assessed using individual *t*-tests. Significance levels are indicated by asterisks (***P < 0.001, **P < 0.01, *P < 0.05, ns *P* ≥ 0.05)

In light of this variation across our diverse data set, we next ask whether our conclusion that an accelerating mortality model (Gompertz) is superior to a constant mortality model also holds when we analyze data within each commonly used host and pathogen taxonomic grouping. In Fig. 3A, we examine ∆*_CG_* within major host taxa (from plants to diverse animal hosts) and find significant support within most of the groupings (i.e., average ∆*_CG_* > 0 per group), favoring the accelerating risk model across the diversity of our experimental host models. In Fig. 3B, we look at the same data from the pathogen perspective and similarly find robust support for accelerating mortality, regardless of whether the pathogen is viral, bacterial, or eukaryotic. Note that although the overall trend agrees with the accelerating mortality hypothesis, not all specific host and pathogen groups show a statistically significant difference between the accelerating and constant mortality models. The underlying AICc scores for all alternate models are presented for each taxonomic grouping of hosts and pathogens in Fig. S2 and S3. In Fig. 3C and B, we further illustrate the broad presence of mortality acceleration across taxonomic groups by plotting the average Gompertz parameter b (accelerating mortality when b>0).

**Fig 4:**
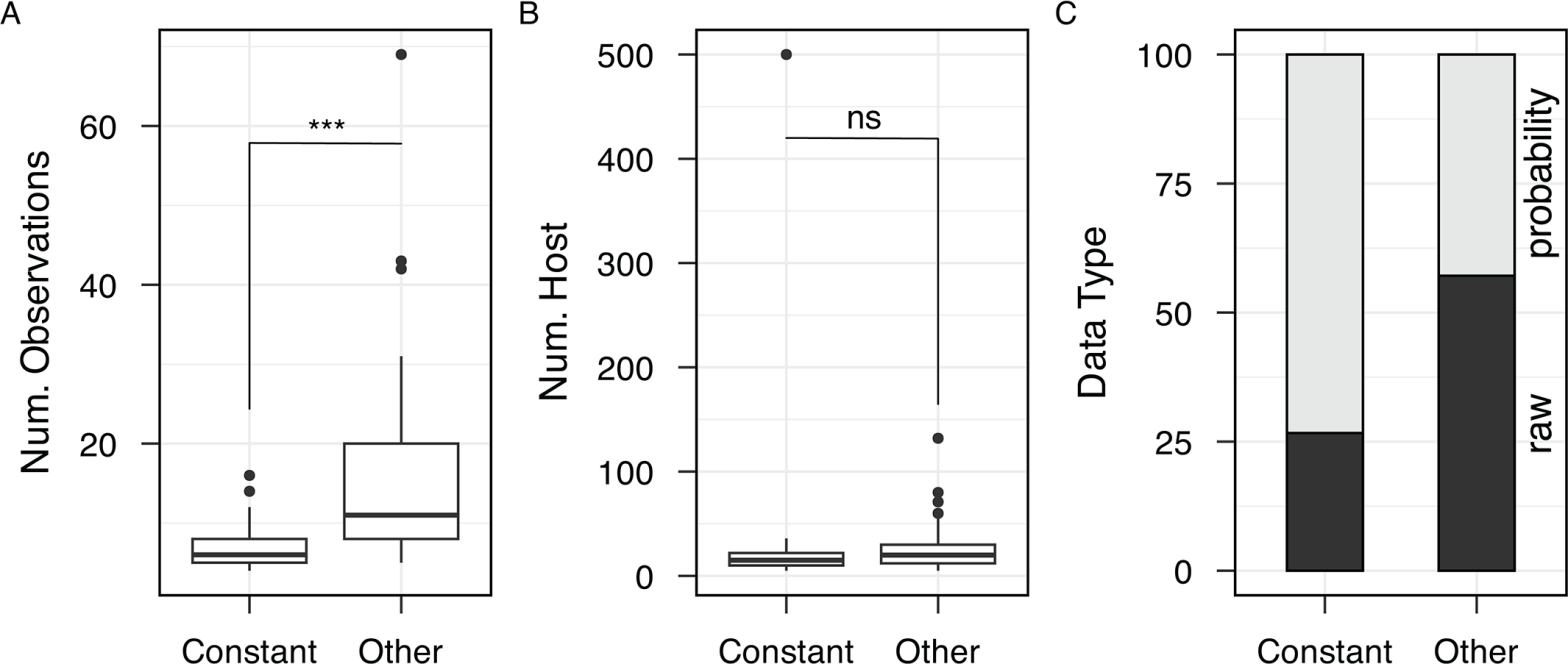
Constant mortality data sets have fewer timepoint observations. Properties of the 25 “constant mortality” experiments are contrasted against the 184 non-constant mortality studies (labeled “other”). Specifically, we contrast (**A**) the number of temporal observations, (**B**) the number of host animals (or plants), and (**C**) the use of raw vs probability data. Asterixes indicate significant differences: (A) two-sample *t*-test, *t =* −7.382*,* df *=* 86.064*, P <* 0.001; (B) two-sample *t*-test, *t* = 0.562, df = 24.197, *P*-value = 0.579; (C) Pearson’s chi-squared test, *X^2^ = 3.135, P =* 0.08.

Figure 3 illustrates that for each taxonomic grouping, the Gompertz model with accelerating mortality is generally superior to the constant mortality model across diverse host and parasite systems. Across all experimental models, we find only 25 out of 209 experimental data sets where the constant mortality model is superior (Fig. 2A). These 25 experiments (see Table S3) raise the question of whether these studies share particular properties, for example, smaller data sets or greater use of probability-based data. In Fig. 4, we show the study properties for the 25 “constant” mortality experiments vs the remaining experiments, illustrating that these experiments have significantly fewer observational time points. This pattern indicates that our conclusion in favor of constant mortality for these 25 studies is likely contingent on limitations in the underlying data sets.

### Connecting mortality to pathogen dynamics and mechanisms of host death

The results in Fig. 1 to 3 show that the extra parameter in the Gompertz model is justified by improved model fit (positive ∆*_CG_*) across a diverse range of experimental infection studies. This supports the conclusion that the risk of death is accelerating approximately exponentially following the initiation of infection—analogous to the acceleration in mortality across entire lifespans, known as aging.

While experimental infection and aging give rise to phenomenologically similar survival patterns, there is a difference in the underlying process. In the case of the experimental infection models illustrated above, we have a clear experimental cause of death—the pathogen. This causal clarity offers a window into the study of mortality, as we have a potential internal currency (pathogen dynamics) to map onto the changing risk of death. In the following sections, we evaluate qualitatively distinct alternate within-host population models for the underlying processes of pathogen growth and host/pathogen interaction and flag the problem of under-determination (37): multiple alternate population models can produce the same phenomenological pattern of exponential increasing instantaneous mortality.

### Deterministic pathogen growth models

The connections between within-host processes of pathogen growth and epidemiological processes of host mortality and transmission have been developed mathematically under the banner of “nested epidemiological models.” Nested models typically take a conventional compartmental susceptible-infected epidemiological model (38) and then attempt to derive epidemiological parameters [mortality $m\left(t\right)$ transmission $\beta \left(t\right)$] as functions of within-host processes of pathogen growth and immune interactions in time t since infection (39, 40).

On a within-host scale, nested epidemiological models can make a range of assumptions on interactions between pathogens, host target cells, and immune effectors (41–44). In simpler models, pathogen reproduction is only regulated by explicit immune dynamics so that pathogen growth is exponential in the absence of effective immune control (as in the case of acute lethal infections) (45, 46). To link pathogen dynamics to host mortality, the most common assumption is that host mortality $m\left(p(t)\right)$ is linearly proportional to pathogen density, $p\left(t\right),$ although other assumptions are sometimes made through empirical evidence (42, 47–49).

Putting these assumptions together, we can describe within-host pathogen density $p\left(t\right)$ as growing exponentially, $p\left(t\right)\;=\;p_{0}\;\cdot \;e^{rt}$, with dynamics governed by initial inoculum $p_{0}$ and a pathogen growth rate r. Next, we assume that instantaneous mortality $m\left(p\right)$ is linearly proportional to the bacterial burden p, with mortality coefficient v, that is, m(t)=v ⋅ p(t). Putting these pieces together, we arrive at an instantaneous mortality function that is driven by pathogen demography and is identical to the Gompertz function (given, $a=v\;\cdot \;p_{0}$ and b=r); $m\left(t\right)\;=\;v\;\cdot \;p_{0}\;\cdot \;e^{rt}$.

While the model assumptions of exponential growth and a linear mapping appear plausible, and the resulting Gompertz equation fits the data above, we must be cautious that multiple mechanistic processes can be consistent with a single statistical pattern (37). To illustrate this simple yet important point, we note that alternate deterministic models can arrive at the same Gompertz endpoint. For example, if pathogen dynamics are linear [$p\left(t\right)=gt$] and the mortality function m(p) is exponential [m(p(t))= ${ve}^{p(t)},$ i.e., higher pathogen burdens are disproportionately damaging], then together these functions again yield Gompertz dynamics $m\left(t\right)={ve}^{gt}$. To further underline this simple argument, we end with an alternate stochastic model with very different assumptions that can produce a very similar phenomenological pattern of accelerating risk of death.

### Stochastic cumulative damage models

While nested models of infectious disease dynamics most commonly rely on deterministic models (50), the aging literature commonly analyzes stochastic models of accelerating death (51). Next, we take a stochastic model of all-causes aging and show how this model can be interpreted in the specific context of infection-mediated death and produces exponentially increasing mortality.

We begin with the assumption that lethal pathogens exert discrete damage events on their host, independent of pathogen burden. We next assume that pathogen-induced damage can be repaired at a limited rate, and this rate of repair declines linearly in time since infection. Finally, we assume that the risk of dying is proportional to the probability that the cumulative “damage queue” exceeds a threshold. These assumptions are consistent with the mathematical model of aging developed by reference 51 and can be analyzed using the theory of queues (52), treating the degree of cumulative damage (the length of the queue) as a stochastic process. The analysis detailed by Ledberg demonstrates that these simple assumptions can also produce an exponential increase in the rate of instantaneous mortality (51), highlighting again that multiple distinct mechanistic processes can generate the same phenomenological pattern of accelerating risk of infection-induced death.

## DISCUSSION

Our data analysis shows that the instantaneous mortality rate increases approximately exponentially during infection in diverse experimental disease models. This exponential increase in risk is a hallmark of aging in humans (27) and other organisms (53) and can be phenomenologically described by the two-parameter Gompertz equation (Table 1). Unlike the aging literature, however, we have a clear causal currency for acute lethal infections. Building on this causal connection, we next outline potential mechanistic paths between the within-host dynamics of the pathogen and the observed acceleration in mortality, using both deterministic and stochastic models. Our analysis demonstrates that multiple causal processes are consistent with the observed survival data, illustrating that inferring within-host behavior purely from mortality trajectories is an under-determined problem. This implies that to connect our epidemiological conclusions of exponentially increasing mortality to underlying mechanisms of pathogen expansion and host damage, we must look inside hosts to observe pathogen dynamics directly.

To address the challenge of measuring within-host pathogen dynamics p(t) , two broad paths are available. The first and most straightforward path is destructive sampling, where a large cohort is tracked during infection, and sample individuals are taken at intervals and sacrificed to estimate pathogen burden at time t, e.g., by grinding up the entire host or a sample of host tissue and plating on selective media for a defined bacterial pathogen (54, 55). Biancalani and Gore used this method to characterize the dynamics of pathogen growth within cohorts of infected nematodes and reported support for a pathogen logistic growth model (54). Yet their protocols illustrate a potential limitation with destructive sampling, namely introducing a selection bias into the data, given the measurement of infection burdens from live worms only. If worms tend to die at high infection burdens, then the apparent plateau in reported bacterial densities could result from the selective removal of worms with high infections due to death.

A second path is to track infection burden non-destructively via repeat measures from the same population of hosts. While requiring fewer hosts to produce workable data (by allowing repeat observations from the same individuals), the requirement to minimize invasive observational effects can become a limitation (56). Fluorescent microscopy and other *in vivo* imaging techniques, such as bioluminescence imaging (BLI) and multiphoton microscopy, allow for non-invasive, real-time monitoring of biological processes in live animals, significantly reducing the need for animal sacrifice (57–60). These are attractive routes, though, in practice, most existing methods are more concerned with pathogen localization and describing disease severity rather than attempting to estimate absolute pathogen densities quantitatively (58, 61). The increased sensitivity and precision offered by sequence-based techniques (e.g., ddPCR) provide the potential for more quantitative measurements although challenges remain over the degree to which a bio-sample is representative of a broader infection context (e.g., see the debate over profiling sputum samples vs more invasive sample collection in people with cystic fibrosis [62]).

Regardless of whether the infection burden is tracked destructively or non-destructively, an important consideration is the extent to which the experimental infection model recapitulates conditions experienced in natural infections. Our inclusion criteria were designed to avoid some obvious “non-natural” conditions, e.g., excluding studies involving genetic modifications of the host or the parasite or the use of treatment interventions, yet there are many other ways in which experimental infection models can depart from conditions in natural (including human) infections (63). Among these possible avenues of departure, we flag our requirement for at least 80% mortality, potentially biasing our experimental data sets toward studies with unrealistically high inocula to rapidly overwhelm host defenses. This requirement was consistent with our focus on lethal infections but leaves open important questions on how host survival and pathogen dynamics interact across a range of inoculum conditions. Duneau et al. made an important step in this direction by assessing how *Drosophila* survival vs a range of bacterial pathogen challenges is dependent on variation in the initial host immune response (55).

This paper focused on diverse experimental infection models with divergent pathogen replication and host immune control modes. Despite this biological diversity, we witnessed a similar phenomenological pattern of accelerating risk. Our approach raises the potential for incorporating age of infection m(t) , and pathogen dynamics $p\left(t\right)$ into epidemiological analyses, a topic pursued under the banner of “nested” epidemiological models (23, 41–43, 64). While previous work on nested epidemiology has relied on “plausible” models of $p\left(t\right)$, e.g., (46, 65), we caution that multiple within-host processes can be consistent with epidemiological data. Our results show the importance of combining direct measures of within-host dynamics of infection with population-scale measures of host mortality. With this combined data, we can constrain models further and explore new avenues for intervention strategies based on the progressive state of infectious disease.

## MATERIALS AND METHODS

After obtaining or digitalizing survival data using WebPlotDigitizer, we performed all the data manipulation, visualization, model fitting, and statistical analysis in R version 4.4.0 (18). Prior to the analysis, we standardized all the survival data and time information to a range of 0 to 1. The final timepoint (proportional time = 1) is defined by the final recorded death event (proportional survival = 0). This standardization ensures comparability across different data sets and facilitates the application of statistical models. Moreover, it allows comparisons of parameter values across data sets. We fitted survival functions described in Table 1 to the standardized data using the nlsLM function from the minipack.lm package in R (17). This function employs the Levenberg-Marquardt nonlinear least squares algorithm to estimate the parameter with initial parameter values (exponential λ=0.1; Gompertz a and b=0.1; Weibull λ=1, k=0.1; log-logistic α=1, β=1; generalized-gamma α=1, β=1, γ=1), and a maximum of 1,000 iterations for convergence control to ensure that the fitting process will stop after a reasonable number of attempts (Fig. S1). Predictive accuracy was evaluated using the corrected Akaike information criterion (AICc). AICc is particularly useful for comparing models on a defined data set, both the goodness of fit and model complexity (number of parameters), and adjusts for small sample sizes (32). To make specific model comparisons within each individual experimental data set, we assess differences in model performance for models *i* and *j* in each study as follows: ∆*_ij_* = AICc*_i_* – AICc*_j_*, where a positive ∆*_ij_* indicates greater support for model *j*, compared to model *i*. Through the later parts of the paper, we focus on the comparison of the Gompertz model (*G*) and constant mortality model (*C*): ∆*_CG_* = AICc*_C_* – AICc*_G_*, where positive values of ∆*_CG_* indicate support for the Gompertz model. We also reported the Gompertz parameter b (b>0 supports accelerating mortality). We run individual *t*-tests (*t-*test function with μ=0, significance threshold *P* < 0.05) to demonstrate whether ∆*_CG_* and *b* are significantly above zero across taxa. All model fits, raw AICc values, and multiple comparisons are reported in Fig. S1 to S4 and Table S1. Given that each data set contributes AICc values for all models, we accounted for non-independence by implementing linear mixed-effects models (LMMs) with Data set as a random effect using the lmer function in lme4 package. This approach appropriately models within-dataset correlations while allowing for general comparisons across models. To test for significant differences in model fits, we conducted type III ANOVA with Satterthwaite’s approximation for degrees of freedom. Post-hoc pairwise comparisons between models were performed using estimated marginal means (emmeans function) with Bonferroni correction to control for multiple comparisons. To further validate our findings, we performed bootstrap resampling (*R* = 1,000) by resampling datasets with replacement and refitting the LMM on each iteration using boot function. The bootstrapped estimates and 95% confidence intervals (CIs) were extracted from the fixed-effect terms of the model. If the bootstrapped CIs excluded zero, we interpreted the comparison as statistically robust. Lastly, LLMs and marginal means are used for assigning compact letter displays (CLDs) using cld function to visually indicate significant groupings for taxa comparisons.

## Data Availability

All individual data sets and data analysis can be accessed at https://github.com/GaTechBrownLab/metaAnalysis.

## SUPPLEMENTAL MATERIAL

Supplemental Figures and Tables msphere.00953-24-s0001.pdf Supplemental figures, Table S1, and captions for Tables S2 and S3.

Table S2 msphere.00953-24-s0002.csv List of papers.

Table S3 msphere.00953-24-s0003.csv Attributes, AIC scores, and parameters.

ASM does not own the copyrights to Supplemental Material that may be linked to, or accessed through, an article. The authors have granted ASM a non-exclusive, world-wide license to publish the Supplemental Material files. Please contact the corresponding author directly for reuse.
