# Supplementary material for: Risk of death during acute infection is accelerating across diverse host-pathogen systems and consistent with multiple models of host-pathogen interaction
Source: mSphere. 2025 Apr 28;10(5):e00953-24. doi: 10.1128/msphere.00953-24 (PMC12108055; doi:10.1128/msphere.00953-24)
Supplement: Supplemental Figures and Tables — Supplemental figures, Table S1, and captions for Tables S2 and S3. [file msphere.00953-24-s0001.pdf]

Supplementary Figures and Tables

Figure S1 (following nine pages)**:** All model fits for each individual dataset.

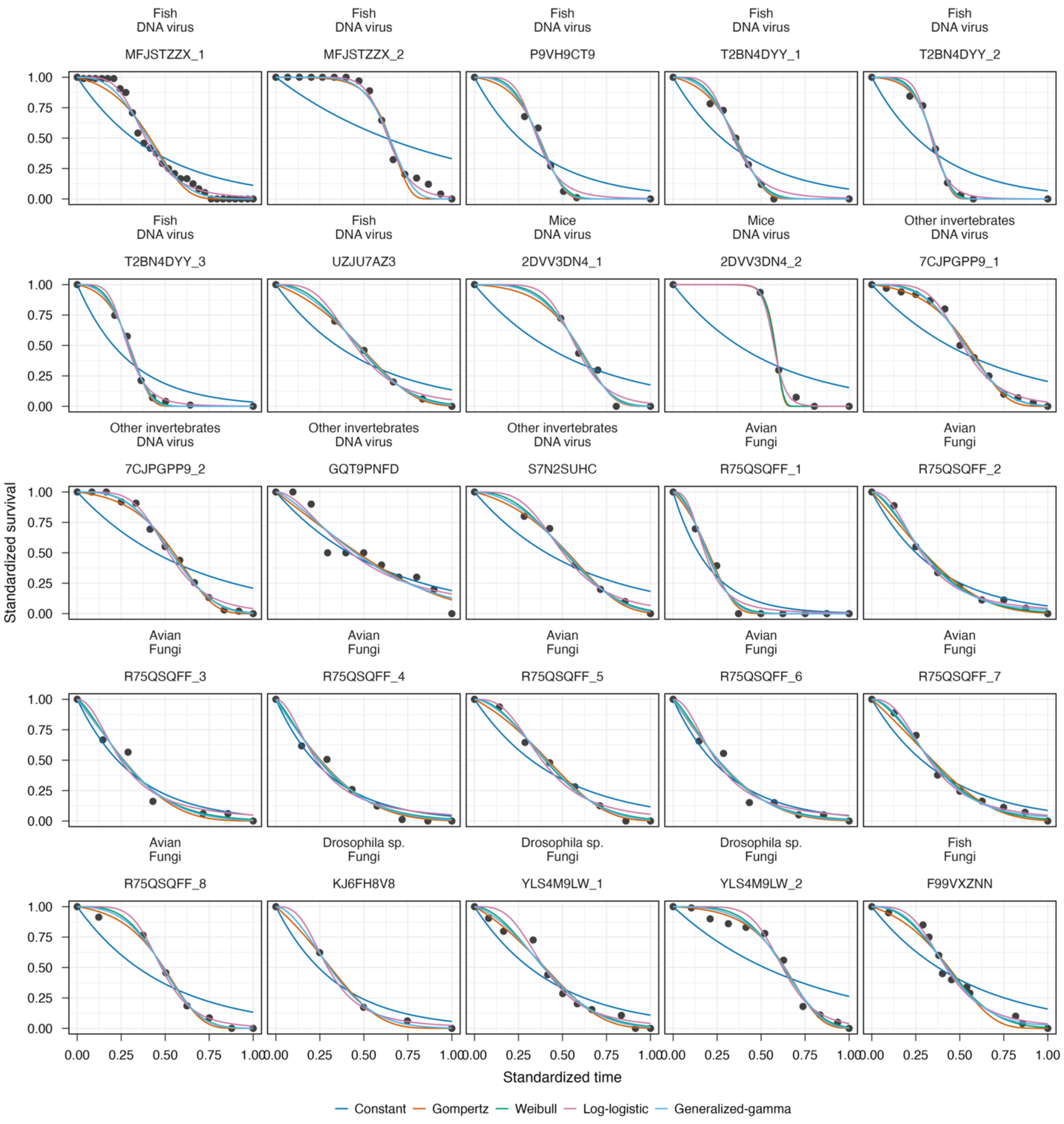

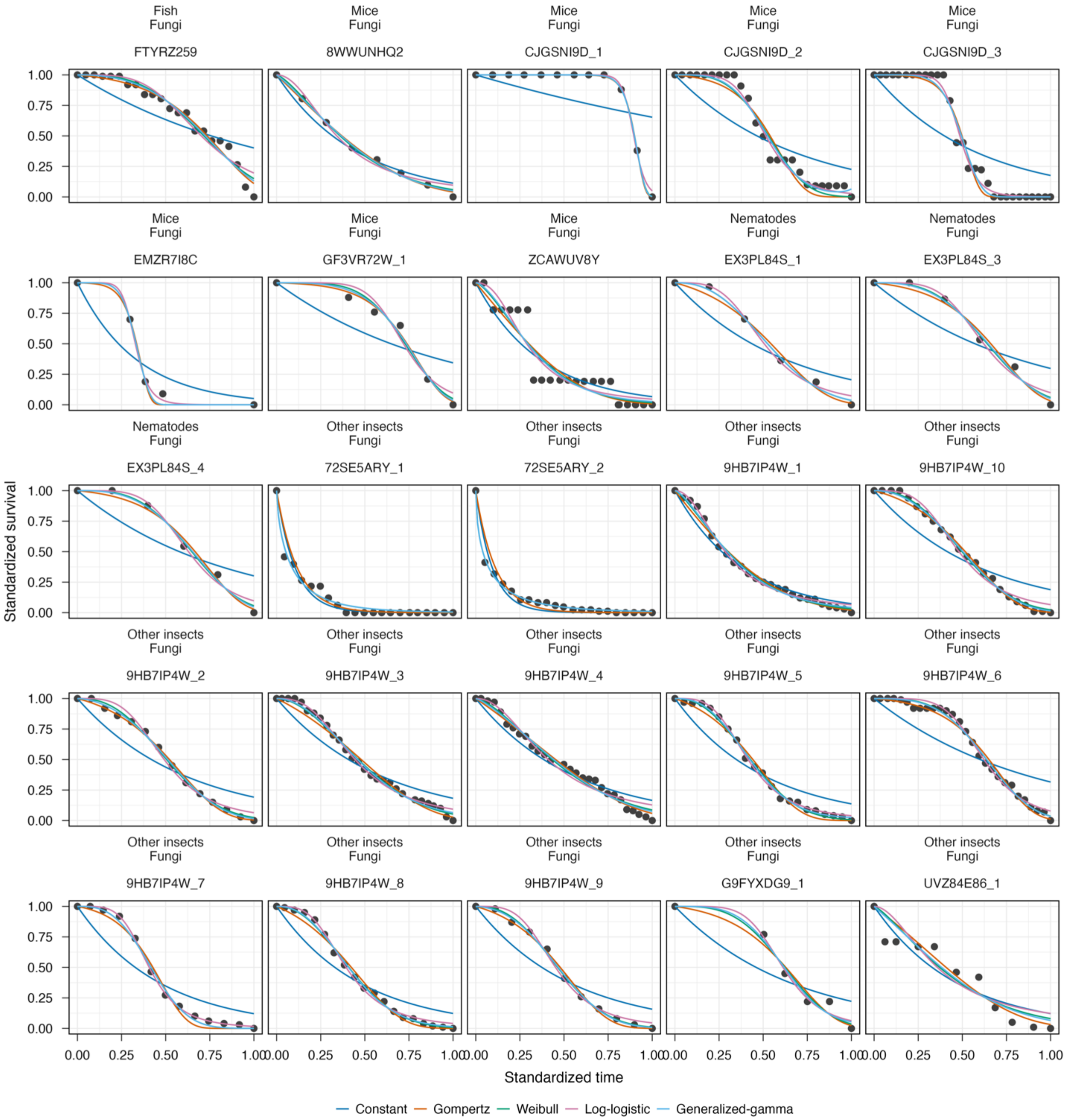

Fish Fungi FTYRZ259
Mice Fungi 8WWUNHQ2
Mice Fungi CJGSNI9D_1
Mice Fungi CJGSNI9D_2
Mice Fungi CJGSNI9D_3
Mice Fungi EMZR7I8C
Mice Fungi GF3VR72W_1
Mice Fungi ZCAWUV8Y
Nematodes Fungi EX3PL84S_1
Nematodes Fungi EX3PL84S_3
Nematodes Fungi EX3PL84S_4
Other insects Fungi 72SE5ARY_1
Other insects Fungi 72SE5ARY_2
Other insects Fungi 9HB7IP4W_1
Other insects Fungi 9HB7IP4W_10
Other insects Fungi 9HB7IP4W_2
Other insects Fungi 9HB7IP4W_3
Other insects Fungi 9HB7IP4W_4
Other insects Fungi 9HB7IP4W_5
Other insects Fungi 9HB7IP4W_6
Other insects Fungi 9HB7IP4W_7
Other insects Fungi 9HB7IP4W_8
Other insects Fungi 9HB7IP4W_9
Other insects Fungi G9FYXDG9_1
Other insects Fungi UVZ84E86_1
Standardized survival
Standardized time
Constant
Gompertz
Weibull
Log-logistic
Generalized-gamma

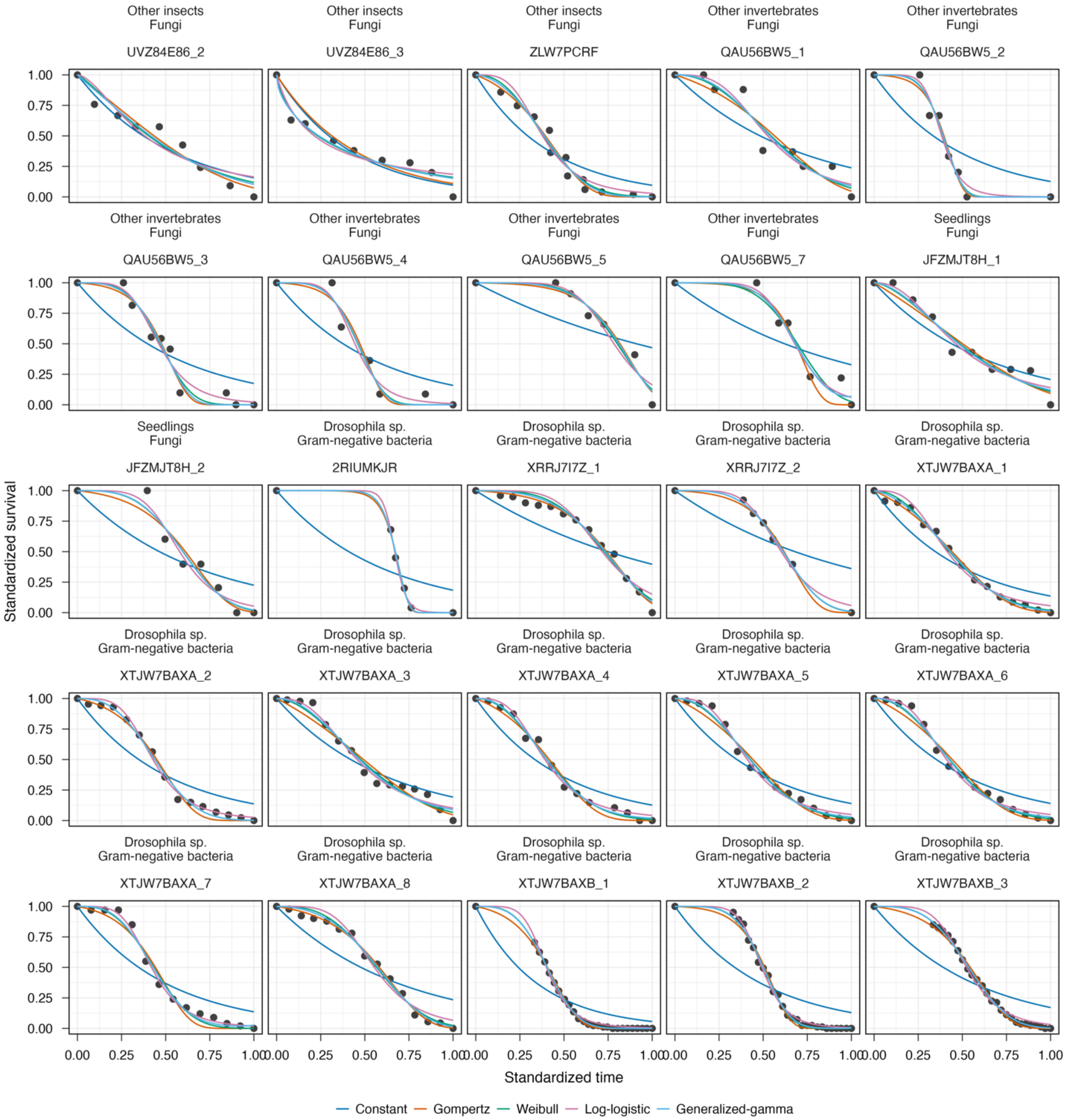
Other insects
Fungi
UVZ84E86_2
Other insects
Fungi
UVZ84E86_3
Other insects
Fungi
ZLW7PCRF
Other invertebrates
Fungi
QAU56BW5_1
Other invertebrates
Fungi
QAU56BW5_2
Other invertebrates
Fungi
QAU56BW5_3
Other invertebrates
Fungi
QAU56BW5_4
Other invertebrates
Fungi
QAU56BW5_5
Other invertebrates
Fungi
QAU56BW5_7
Seedlings
Fungi
JFZMJT8H_1
Seedlings
Fungi
JFZMJT8H_2
Drosophila sp.
Gram-negative bacteria
2RIUMKJR
Drosophila sp.
Gram-negative bacteria
XRRJ7I7Z_1
Drosophila sp.
Gram-negative bacteria
XRRJ7I7Z_2
Drosophila sp.
Gram-negative bacteria
XTJW7BAXA_1
Drosophila sp.
Gram-negative bacteria
XTJW7BAXA_2
Drosophila sp.
Gram-negative bacteria
XTJW7BAXA_3
Drosophila sp.
Gram-negative bacteria
XTJW7BAXA_4
Drosophila sp.
Gram-negative bacteria
XTJW7BAXA_5
Drosophila sp.
Gram-negative bacteria
XTJW7BAXA_6
Drosophila sp.
Gram-negative bacteria
XTJW7BAXA_7
Drosophila sp.
Gram-negative bacteria
XTJW7BAXA_8
Drosophila sp.
Gram-negative bacteria
XTJW7BAXB_1
Drosophila sp.
Gram-negative bacteria
XTJW7BAXB_2
Drosophila sp.
Gram-negative bacteria
XTJW7BAXB_3
Standardized survival
Standardized time
Constant
Gompertz
Weibull
Log-logistic
Generalized-gamma

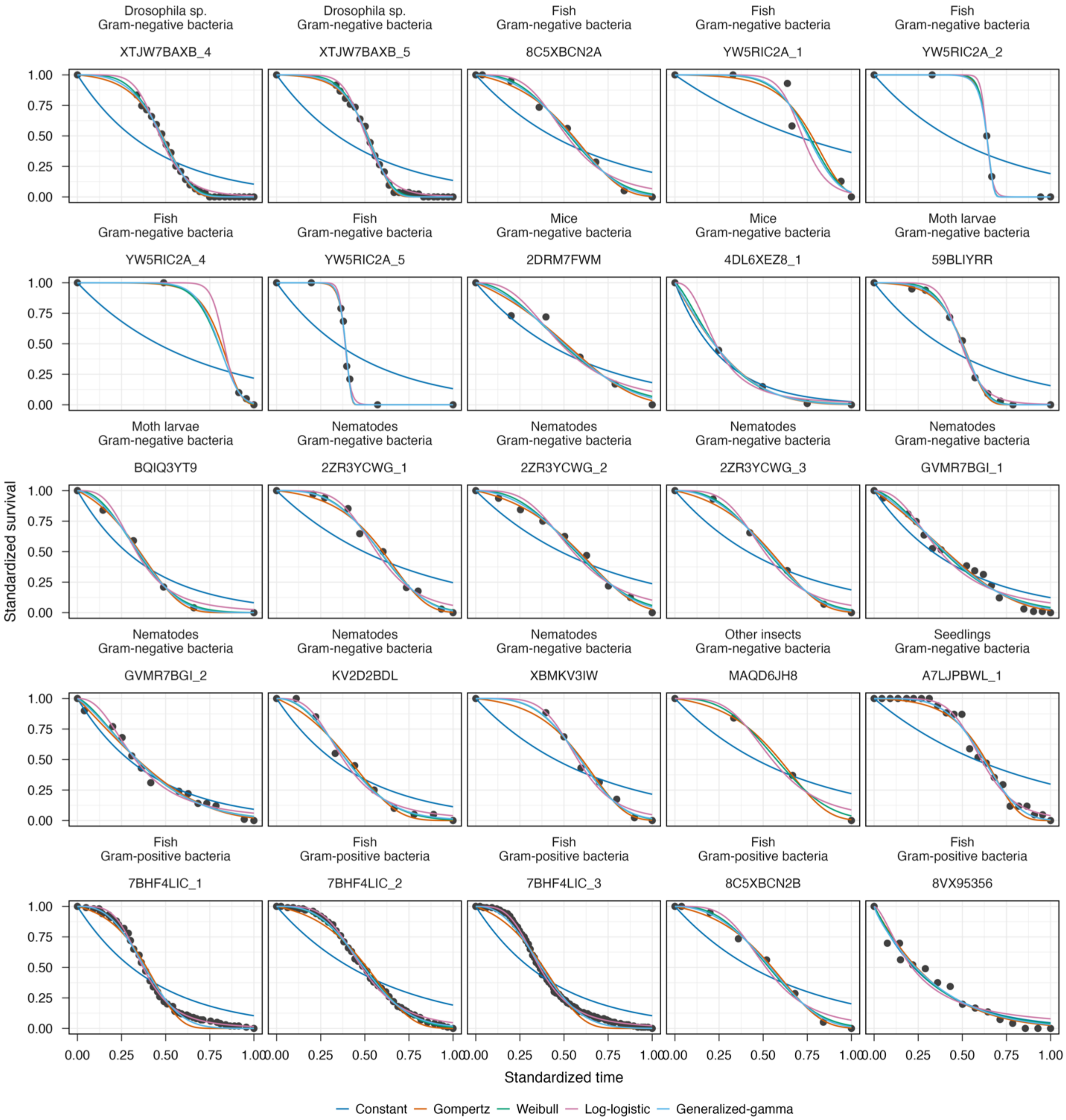

Drosophila sp.
Gram-negative bacteria
XTJW7BAXB_4
Drosophila sp.
Gram-negative bacteria
XTJW7BAXB_5
Fish
Gram-negative bacteria
8C5XBCN2A
Fish
Gram-negative bacteria
YW5RIC2A_1
Fish
Gram-negative bacteria
YW5RIC2A_2
Fish
Gram-negative bacteria
YW5RIC2A_4
Fish
Gram-negative bacteria
YW5RIC2A_5
Mice
Gram-negative bacteria
2DRM7FWM
Mice
Gram-negative bacteria
4DL6XEZ8_1
Moth larvae
Gram-negative bacteria
59BLIYRR
Moth larvae
Gram-negative bacteria
BQIQ3YT9
Nematodes
Gram-negative bacteria
2ZR3YCWG_1
Nematodes
Gram-negative bacteria
2ZR3YCWG_2
Nematodes
Gram-negative bacteria
2ZR3YCWG_3
Nematodes
Gram-negative bacteria
GVMR7BGI_1
Nematodes
Gram-negative bacteria
GVMR7BGI_2
Nematodes
Gram-negative bacteria
KV2D2BDL
Nematodes
Gram-negative bacteria
XBMKV3IW
Other insects
Gram-negative bacteria
MAQD6JH8
Seedlings
Gram-negative bacteria
A7LJPBWL_1
Fish
Gram-positive bacteria
7BHF4LIC_1
Fish
Gram-positive bacteria
7BHF4LIC_2
Fish
Gram-positive bacteria
7BHF4LIC_3
Fish
Gram-positive bacteria
8C5XBCN2B
Fish
Gram-positive bacteria
8VX95356
Standardized survival
1.00
0.75
0.50
0.25
0.00
Standardized time
0.00 0.25 0.50 0.75 1.00
Constant
Gompertz
Weibull
Log-logistic
Generalized-gamma

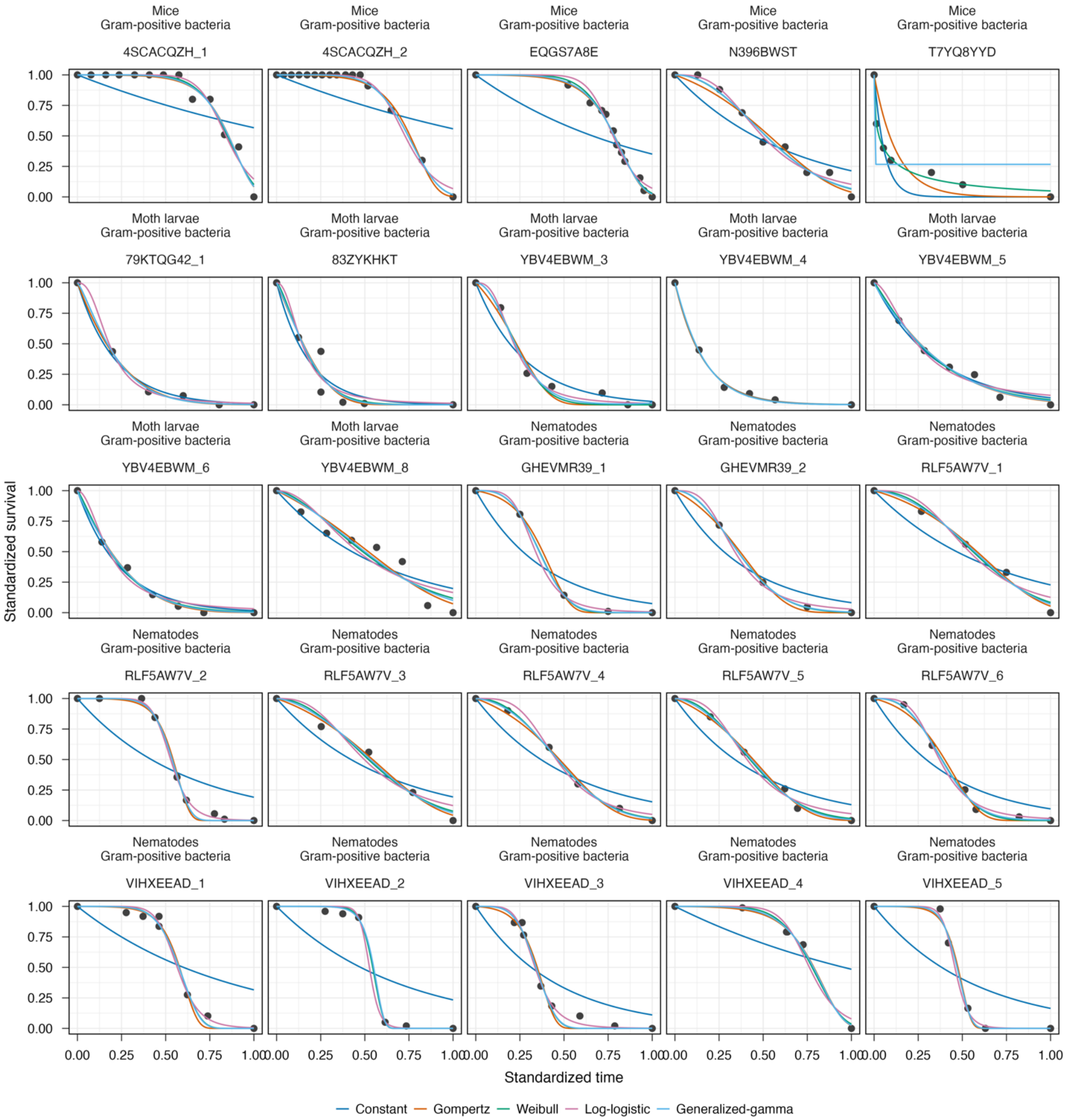

Mice Gram-positive bacteria 4SCACQZH_1
Mice Gram-positive bacteria 4SCACQZH_2
Mice Gram-positive bacteria EQGS7A8E
Mice Gram-positive bacteria N396BWST
Mice Gram-positive bacteria T7YQ8YYD
Moth larvae Gram-positive bacteria 79KTQG42_1
Moth larvae Gram-positive bacteria 83ZYKHKT
Moth larvae Gram-positive bacteria YBV4EBWM_3
Moth larvae Gram-positive bacteria YBV4EBWM_4
Moth larvae Gram-positive bacteria YBV4EBWM_5
Moth larvae Gram-positive bacteria YBV4EBWM_6
Moth larvae Gram-positive bacteria YBV4EBWM_8
Nematodes Gram-positive bacteria GHEVMR39_1
Nematodes Gram-positive bacteria GHEVMR39_2
Nematodes Gram-positive bacteria RLF5AW7V_1
Nematodes Gram-positive bacteria RLF5AW7V_2
Nematodes Gram-positive bacteria RLF5AW7V_3
Nematodes Gram-positive bacteria RLF5AW7V_4
Nematodes Gram-positive bacteria RLF5AW7V_5
Nematodes Gram-positive bacteria RLF5AW7V_6
Nematodes Gram-positive bacteria VIHXEEAD_1
Nematodes Gram-positive bacteria VIHXEEAD_2
Nematodes Gram-positive bacteria VIHXEEAD_3
Nematodes Gram-positive bacteria VIHXEEAD_4
Nematodes Gram-positive bacteria VIHXEEAD_5
Standardized survival
Standardized time
Constant
Gompertz
Weibull
Log-logistic
Generalized-gamma

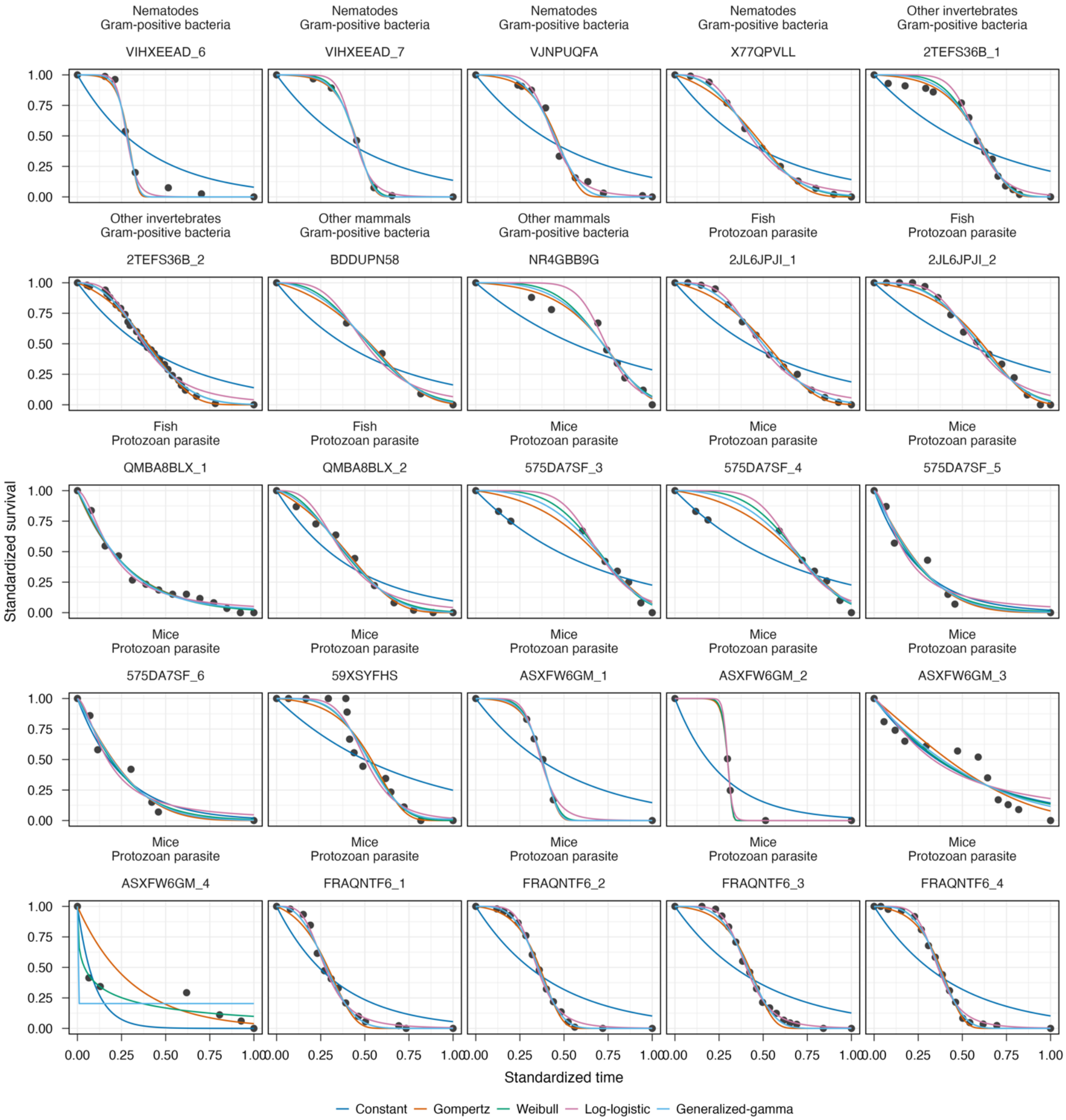

Nematodes Gram-positive bacteria VIHXEEAD_6
Nematodes Gram-positive bacteria VIHXEEAD_7
Nematodes Gram-positive bacteria VJNPUQFA
Nematodes Gram-positive bacteria X77QPVLL
Other invertebrates Gram-positive bacteria 2TEFS36B_1
Other invertebrates Gram-positive bacteria 2TEFS36B_2
Other mammals Gram-positive bacteria BDDUPN58
Other mammals Gram-positive bacteria NR4GBB9G
Fish Protozoan parasite 2JL6JPJI_1
Fish Protozoan parasite 2JL6JPJI_2
Fish Protozoan parasite QMBA8BLX_1
Fish Protozoan parasite QMBA8BLX_2
Mice Protozoan parasite 575DA7SF_3
Mice Protozoan parasite 575DA7SF_4
Mice Protozoan parasite 575DA7SF_5
Mice Protozoan parasite 575DA7SF_6
Mice Protozoan parasite 59XSYFHS
Mice Protozoan parasite ASXFW6GM_1
Mice Protozoan parasite ASXFW6GM_2
Mice Protozoan parasite ASXFW6GM_3
Mice Protozoan parasite ASXFW6GM_4
Mice Protozoan parasite FRAQNTF6_1
Mice Protozoan parasite FRAQNTF6_2
Mice Protozoan parasite FRAQNTF6_3
Mice Protozoan parasite FRAQNTF6_4
Standardized survival
1.00 0.75 0.50 0.25 0.00
Standardized time
0.00 0.25 0.50 0.75 1.00
Constant Gompertz Weibull Log-logistic Generalized-gamma

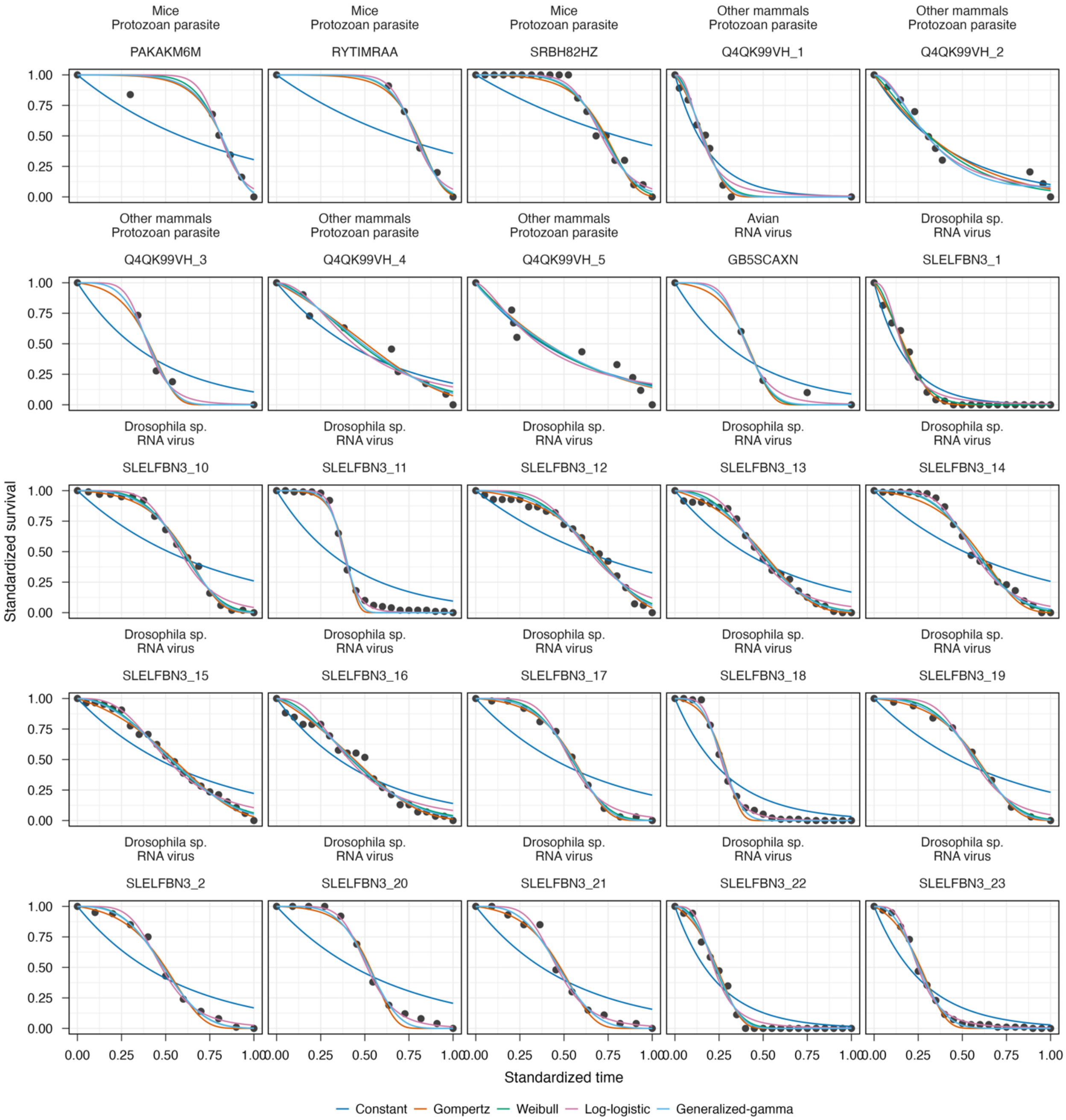

Mice
Protozoan parasite
PAKAKM6M
Mice
Protozoan parasite
RYTIMRAA
Mice
Protozoan parasite
SRBH82HZ
Other mammals
Protozoan parasite
Q4QK99VH_1
Other mammals
Protozoan parasite
Q4QK99VH_2
Other mammals
Protozoan parasite
Q4QK99VH_3
Other mammals
Protozoan parasite
Q4QK99VH_4
Other mammals
Protozoan parasite
Q4QK99VH_5
Avian
RNA virus
GB5SCAXN
Drosophila sp.
RNA virus
SLELFBN3_1
Drosophila sp.
RNA virus
SLELFBN3_10
Drosophila sp.
RNA virus
SLELFBN3_11
Drosophila sp.
RNA virus
SLELFBN3_12
Drosophila sp.
RNA virus
SLELFBN3_13
Drosophila sp.
RNA virus
SLELFBN3_14
Drosophila sp.
RNA virus
SLELFBN3_15
Drosophila sp.
RNA virus
SLELFBN3_16
Drosophila sp.
RNA virus
SLELFBN3_17
Drosophila sp.
RNA virus
SLELFBN3_18
Drosophila sp.
RNA virus
SLELFBN3_19
Drosophila sp.
RNA virus
SLELFBN3_2
Drosophila sp.
RNA virus
SLELFBN3_20
Drosophila sp.
RNA virus
SLELFBN3_21
Drosophila sp.
RNA virus
SLELFBN3_22
Drosophila sp.
RNA virus
SLELFBN3_23
Standardized survival
1.00
0.75
0.50
0.25
0.00
Standardized time
0.00
0.25
0.50
0.75
1.00
Constant
Gompertz
Weibull
Log-logistic
Generalized-gamma

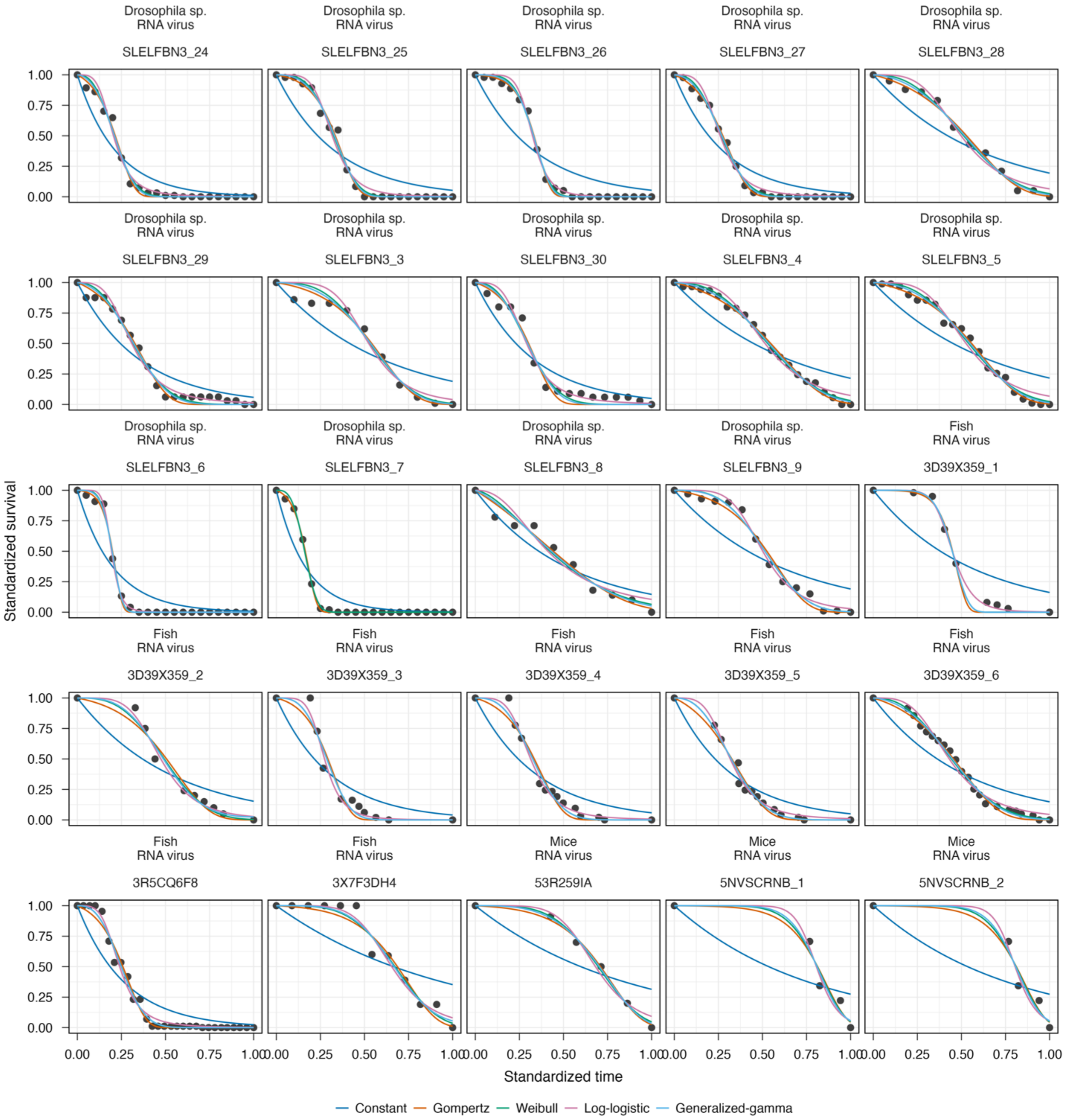

Drosophila sp.
RNA virus
SLELFBN3_24
SLELFBN3_25
SLELFBN3_26
SLELFBN3_27
SLELFBN3_28
SLELFBN3_29
SLELFBN3_3
SLELFBN3_30
SLELFBN3_4
SLELFBN3_5
SLELFBN3_6
SLELFBN3_7
SLELFBN3_8
SLELFBN3_9
Fish
3D39X359_1
3D39X359_2
3D39X359_3
3D39X359_4
3D39X359_5
3D39X359_6
3R5CQ6F8
3X7F3DH4
Mice
53R259IA
5NVSCRNB_1
5NVSCRNB_2
Standardized survival
Standardized time
0.00
0.25
0.50
0.75
1.00
Constant
Gompertz
Weibull
Log-logistic
Generalized-gamma

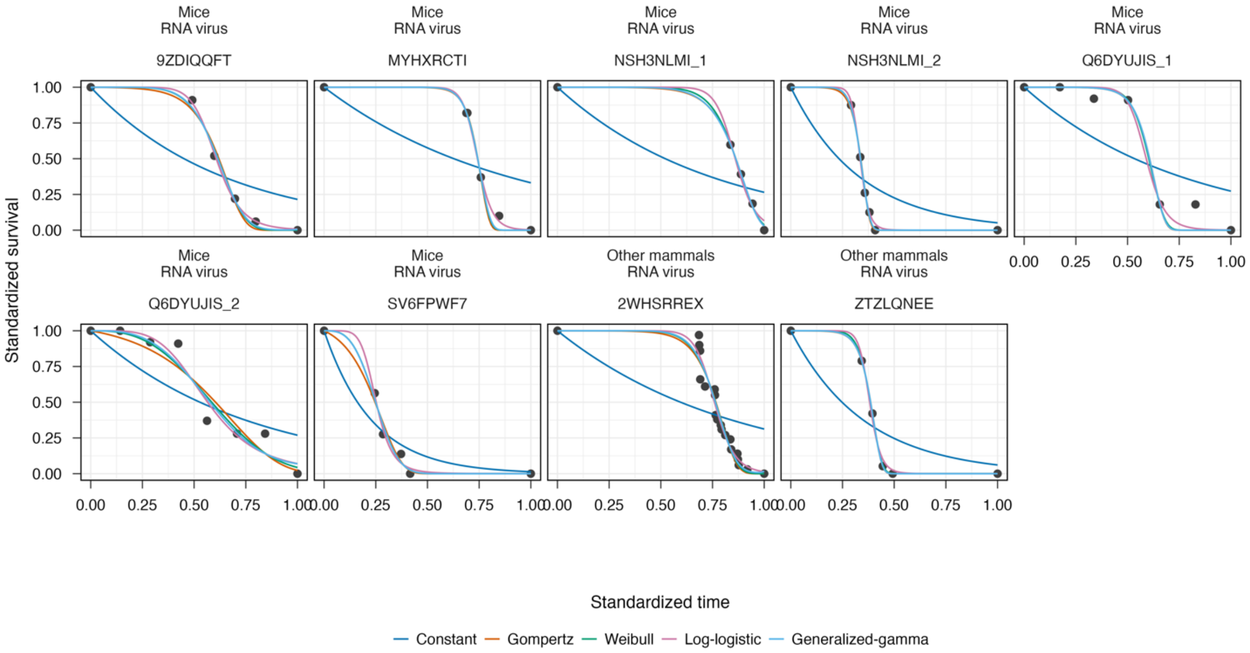

End of Figure S1.

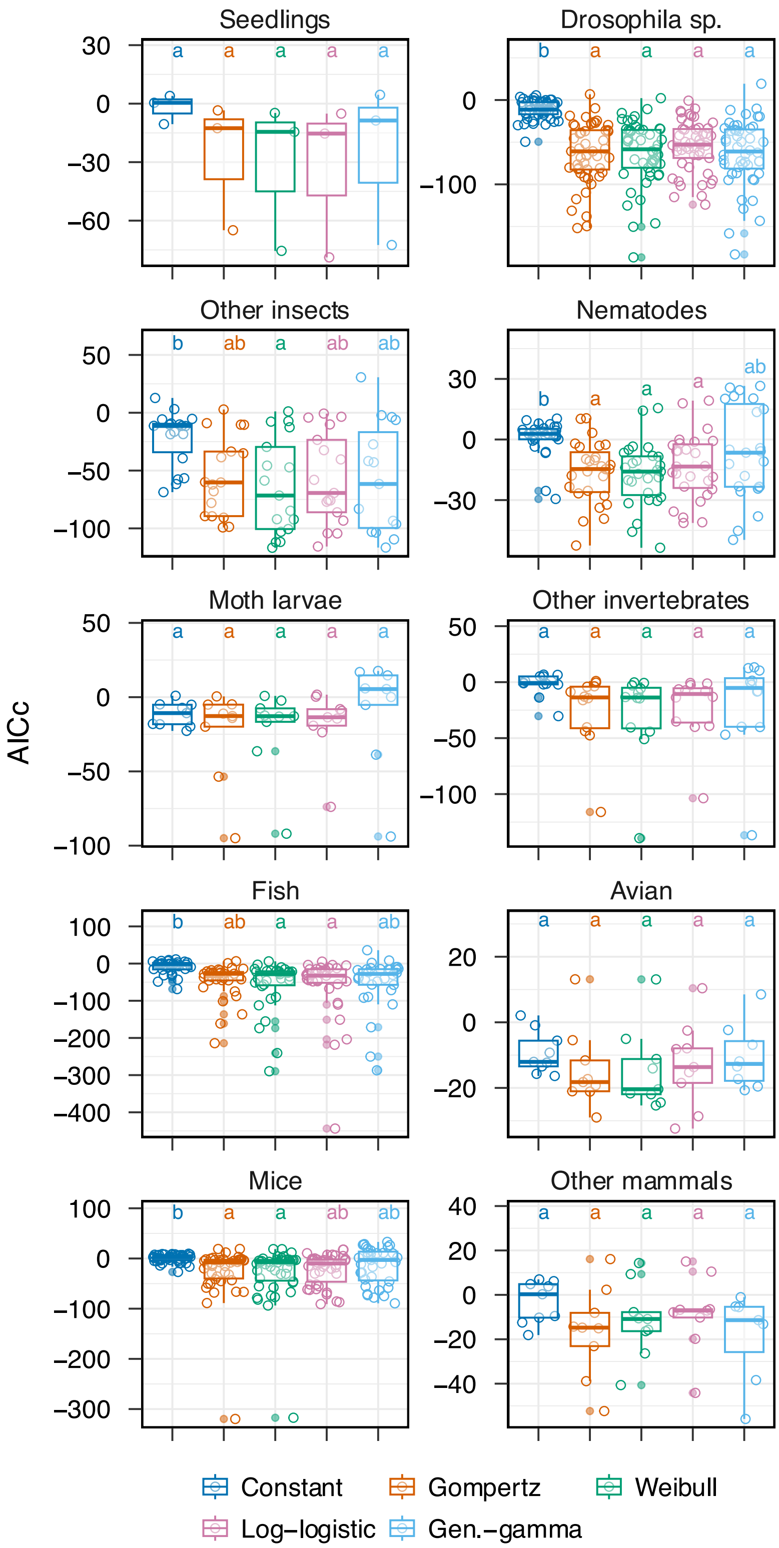

The figure caption is on the next page.

Figure S2: AICc distribution of a specific mortality model for each host category. Points represent individual datasets. Model comparisons for different host categories based on AICc values were analyzed using a linear mixed-effects model (LME) with Dataset as a random effect to account for non-independence of data points. Estimated marginal means (EMMs) were obtained using the emmeans function, and pairwise comparisons were adjusted for multiple testing (Bonferroni correction). Letters above the boxplots indicate statistical groupings based on compact letter display (CLD) derived from pairwise EMM comparisons. Models sharing a letter are not significantly different from each other (*α = 0.05*).

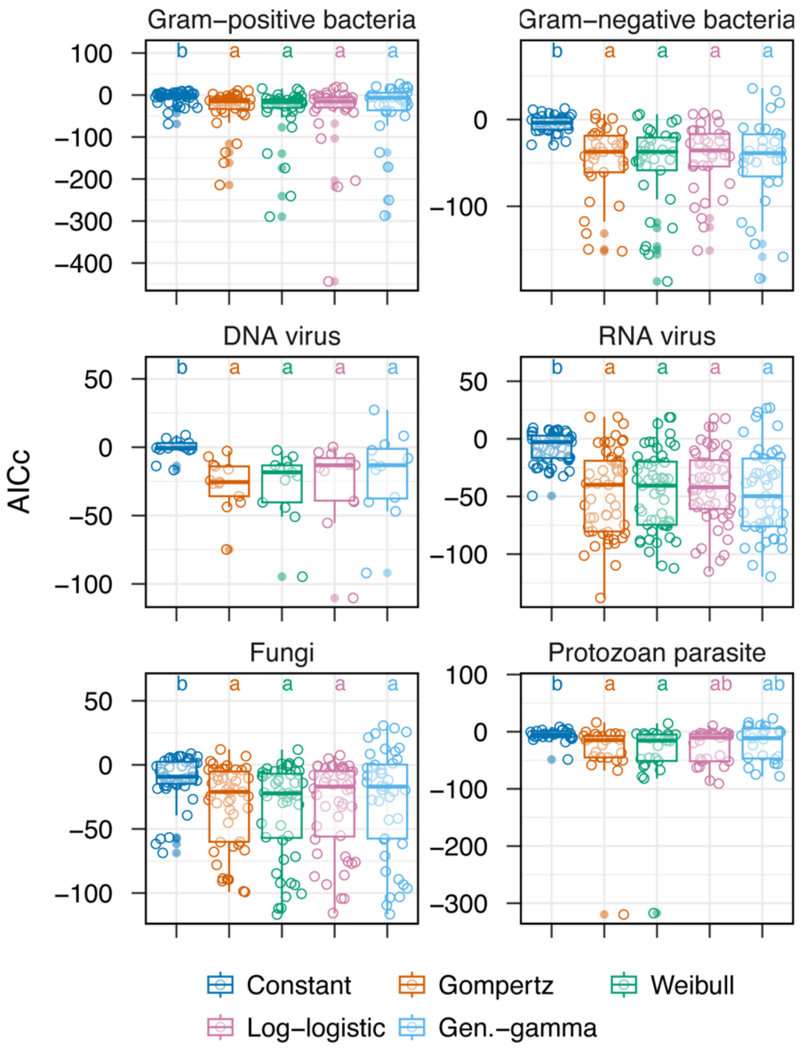

Figure S3: AICc distribution of a specific mortality model for each pathogen category. Points represent individual datasets. Model comparisons for different pathogen categories based on AICc values were analyzed using a linear mixed-effects model (LME) with Dataset as a random effect to account for non-independence of data points. Estimated marginal means (EMMs) were obtained using the emmeans function, and pairwise comparisons were adjusted for multiple testing (Bonferroni correction). Letters above the boxplots indicate statistical groupings based on compact letter display (CLD) derived from pairwise EMM comparisons. Models sharing a letter are not significantly different from each other (*α = 0.05*).

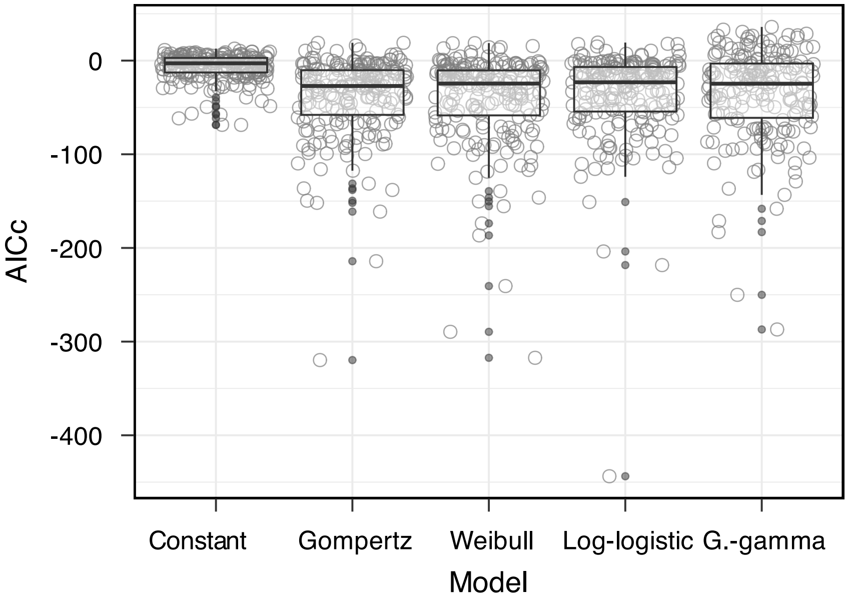

Figure S4: AICc values of all models fitted to all datasets. See Table S1 below for statistical analysis.

Table S1: Pairwise comparisons of AICc values between different models. Estimates displays the estimated differences obtained from the mixed-effects model, Positive values indicate that the second model (right-hand side) has a higher AICc, meaning it fits worse than the first model. SE (model) is the standard error of the estimate. Denominator degrees of freedom (Den. df) were estimated using Satterthwaite's approximation, which accounts for the mixed-effects structure of the model. T-ratio is the test statistic from the mixed model. The significance level of the comparison (*** $p < 0.001$, ** $p < 0.01$, * $p < 0.05$, ns $p \geq 0.05$). Bootstrapped estimates are the mean pairwise difference in AICc values across 1000 bootstrap resamples. SE (bootstrap) is the standard error of the bootstrapped estimates. CI is the 95% confidence interval of the bootstrapped estimate. Non-overlapping confidence intervals with zero indicate strong evidence for differences between models.

	Model estimates					Bootstrapped estimates		
**Contrast**	**Estimate**	**SE**	**Den. df**	**t-ratio**	**Sig. level**	**Estimate**	**SE**	**CI**
Constant–Gompertz	30.918	2.146	811	14.409	***	33.951	2.722	29.026–39.503
Constant–Weibull	33.845	2.146	811	15.774	***	26.742	2.861	21.249–32.516
Constant–Log–logistic	29.283	2.146	811	13.647	***	29.391	2.844	23.826–35.161
Constant–G. gamma	26.760	2.207	812	12.124	***	30.946	2.644	25.859–36.126
Gompertz–Weibull	2.928	2.146	810	1.364	ns	-7.209	1.855	-10.985–3.793
Gompertz–Log-logistic	-1.635	2.146	810	-0.762	ns	-4.560	1.978	-8.366–1.028
Gompertz–G. gamma	4.157	2.208	812	1.883	ns	3.004	1.735	-0.373–6.494
Weibull–Log-logistic	4.563	2.146	810	2.126	ns	-2.649	2.085	-6.527–1.585
Weibull–G. gamma	7.085	2.208	812	3.209	*	-4.205	1.830	-7.836–0.724
Log-logistic–G. gamma	2.522	2.208	812	1.142	ns	-1.555	2.027	-5.428–2.476

Supplementary Data Tables

Supplementary Table 2: TableS2_List_of_Papers.csv. Papers used in this work and keys to the individual datasets.

Supplementary Table 3: TableS3_Attributes_AICc_and_parameters.csv. Dataset attributes, associated metainformation, model parameters, and model accuracy measurements.
